# The Immune Landscape of Canine Soft Tissue Sarcomas as a Model for Human Soft Tissue Sarcomas

**DOI:** 10.3390/cancers17233860

**Published:** 2025-11-30

**Authors:** Regina Hayburn, Dongjun Chung, Arkobato Gupta, Shane Sills, Jennifer Donglan Wu, Andy Ambrus, Weiqing Jing, Juliana Ng, Pablo Penaloza-MacMaster, Aubrie Selmek, Seth M. Pollack, Shay Bracha

**Affiliations:** 1 Department of Clinical Sciences, College of Veterinary Medicine, Texas A&M University, College Station, TX 77840, USA; 2 Department of Biomedical Informatics, The Ohio State University College of Medicine, Columbus, OH 43210, USA; 3 Department of Clinical Sciences, College of Veterinary Medicine, The Ohio State University, Columbus, OH 43210, USA; 4 Robert H. Lurie Comprehensive Cancer Center, Northwestern University Feinberg School of Medicine, Chicago, IL 60611, USAseth.pollack@northwestern.edu (S.M.P.); 5 Department of Hematology and Oncology, Northwestern University Feinberg School of Medicine, Chicago, IL 60611, USA; 6 Center for Virology and Vaccine Research, Beth Israel Deaconess Medical Center, Harvard University Medical School, Boston, MA 02115, USA

**Keywords:** soft tissue sarcoma, undifferentiated pleomorphic sarcoma, canine, tumor microenvironment, immune contexture

## Abstract

**Background/Objectives:** Soft tissue sarcomas (STS) remain a therapeutic challenge due to their limited response to radiation and conventional chemotherapies. While recent advances in immunotherapy have improved outcomes in several cancers, these strategies have been largely disappointing in STS patients. Naturally occurring STS in dogs have been suggested as a spontaneous, immunocompetent model of human STS, but further characterization of its tumor immune microenvironment is needed to validate its relevance. This study aimed to identify the shared immune-related components of canine and human STS and to determine how these factors influence the tumor biology, progression, and prognosis. **Results:** Data from 75 dogs with STS was analyzed. In addition, we characterized the tumor immune microenvironment using immunohistochemistry and compared gene expression between canine and human STS. Progression-free survival and time to metastasis was significantly longer in castrated males in comparison to females. In addition, dogs with appendicular tumors had better progression- and recurrence-free survival, whereas tumor recurrence following surgical excision was associated with a shorter time to metastasis. Immunohistochemistry revealed infiltration of CD204^+^ cells in most of the tumors examined, and disease-free intervals were shorter in dogs with tumors exhibiting FOXP3^+^ cell infiltration. Gene expression profiling demonstrated similarities between canine STS and human undifferentiated pleomorphic sarcomas, with MYC dysregulation emerging as a poor prognostic indicator for dogs. **Conclusions:** The comparative analysis between the human and canine STS microenvironment offers a valuable insight into the clinical behavior and immune landscape of canine STS, underscoring its potential as a relevant preclinical model for the translation and development of future immunotherapies.

## Simple Summary

Soft tissue sarcomas (STS) are a broad group of aggressive connective tissue tumors that affect dogs and humans. This study aimed to compare STS between the two species and identify the shared characteristics of their immune contextures. We analyzed clinical data, tumor histopathology, and gene expression profiles from STS from 75 dogs, and compared them to human STS. Our results revealed that castrated male dogs had worse progression-free survival and shorter time to metastasis compared to spayed females, suggesting that factors other than sex hormones determine patients’ outcome. In addition, canine STS tumors exhibited immune contextures that mirrored those observed in human, characterized by significant infiltration of CD204 macrophages. In cases of increased FOXP3^+^ Tregs presence, a significant correlation with shorter progression-free intervals was observed. We also identified similarities in gene expression between canine STS and human undifferentiated pleomorphic sarcoma, with MYC dysregulation in canine indicating poor prognosis. The results of this study highlight the potential of canine STS as a preclinical model for the disease in humans and for advancing the development of novel immunotherapies.

## 1. Introduction

Soft tissue sarcomas (STS) are a diverse group of over 50 different tumors of mesenchymal origin that share similar biological behaviors [1,2,3]. In the United States, more than 13,000 individuals are diagnosed with STS annually, contributing to approximately 1% of all solid tumors [4,5,6,7]. Despite their relatively low incidence, STS pose a significant clinical challenge, as nearly 50% of patients eventually develop metastatic disease. The five-year survival rate for patients with localized STS is approximately 81%; however, for those with distant metastases, it is as low as 18% [8,9]. Histological grade is a well-established prognostic factor that strongly correlates with the risk of metastatic disease [8,9]. For patients with advanced or metastatic STS, palliative chemotherapy remains the mainstay of treatment, with a median survival of approximately 24 months from first diagnosis [8,9].

Immunotherapies have transformed the oncology field, greatly improving outcomes for many patients across several malignancies [10]. However, these successes have not been mirrored in STS patients, largely due to the tumor’s immunosuppressive nature [11,12,13]. Although isolated sarcoma subtypes have been found to have outstanding responses to checkpoint inhibitors and cellular therapy, on-going trials are likely to bring immunotherapy to an even wider range of STS patients [14,15,16]. Advancing our understanding of the immune contexture within the tumor microenvironment (TME) and identifying immunologically relevant STS models are essential for accelerating the development of novel therapies.

Similarly to the disease in human, canine STS encompass a broad spectrum of mesenchymal tumors with comparable biological behavior. Canine STS account for approximately 15% and 7% of all cutaneous and subcutaneous tumors, respectively, with a predilection for the trunk and extremities [16]. These tumors exhibit aggressive local behavior, necessitating surgical resection and, in some cases, adjuvant radiation therapy [16,17]. Residual disease resulting from incomplete surgical removal is associated with higher recurrence rates which correlate with tumor grade, reaching up to 78% in grade 3 tumors [16,17]. The shared biological characteristics between canine and human STS, combined with the higher incidence of STS in dogs, underscore the value of canine models for studying this disease [18,19]. More so, several studies have identified the similarities between the most common human STS subtype, undifferentiated pleomorphic sarcoma (UPS) and related STS-subtypes, with canine STS regarding immune infiltration and morphology by immunohistochemistry [18,19].

The tumor microenvironment of solid tumors is highly complex, consisting of malignant cells, stromal components, vasculature, extracellular matrix, and immune cells [20,21,22]. The proliferation of tumor-infiltrating lymphocytes has been associated with favorable outcomes in human STS [23]. Tumors can be categorized as either “hot” or “cold” based on their immune profile. “Hot” tumors are characterized by a substantial presence of immune cell infiltrates within the tumor stroma, whereas “cold” tumors exhibit minimal immune cell infiltration [20,21,22]. Cold tumors often display impaired antigen presentation and a suboptimal adaptive immune response, typically associated with increased expression of inhibitory ligands and reduced expression of costimulatory molecules and class I major histocompatibility complex (MHC) molecules, all of which correlate with poor prognosis in STS patients [21,22,23,24,25,26,27]. In soft tissue sarcomas (STS), the tumor microenvironment is thought to be immunosuppressive, inhibiting anti-tumor immunity by exhibiting low proliferation of cytotoxic T cells and elevated numbers of regulatory T cells [23,24,25,26,27].

The current study will further characterize the similarities between the tumors of both species by comparing the gene expression signature in a large cohort of dogs and human patients. Given the great need for naturally occurring and immunocompetent models, a detailed investigation and comparison of the tumor immune microenvironment between the two species is warranted for advancing our understanding for future comparative studies.

## 2. Materials and Methods

### 2.1. Study Population

Dogs with a histological diagnosis of soft tissue sarcoma (STS) were identified by an electronic medical record search from Texas A&M University Veterinary Teaching Hospital between June 2011 and November 2021. Patients that met the inclusion criteria were dogs with a histologic diagnosis of STS who underwent surgical excision of the mass for primary removal. Cases were excluded if no histopathologic diagnosis was obtained or if the primary lesion was excised prior to referral for clinical staging and adjunctive therapy. The following clinical information was recorded upon retrospective review of electronic medical records: patient signalment, date of initial presentation, diagnostic imaging interpretation, primary lesion size and location, disease stage, date of surgery, surgical site infections, and date of disease progression where applicable. Disease progression was determined from the medical record and was one of the following: identification of a new lesion, confirmation of local recurrence in the surgical site, or metastatic disease. Dates of therapeutic interventions were recorded for all patients. Tissue samples were evaluated by board-certified anatomic pathologists and reports reviewed for documentation of histologic diagnosis.

### 2.2. Immunohistochemistry

The immunohistochemical assay was performed on an intelliPATH Flex automated stainer (Biocare Medical, Pacheco, CA, USA). Sections of soft tissue sarcomas were cut at 4 um and mounted on charged slides. The slides were deparaffinized in xylene and rehydrated in graded alcohols. The processed slides were placed in a programable pressure cooker (Decloaking chamber, Biocare Medical) with a pH 6.2 retrieval buffer. After antigen retrieval, endogenous peroxidase was blocked with 3% hydrogen peroxide and non-specific protein binding was blocked with Background Punisher (Biocare Medical). The slides were then incubated for 30 min with the following primary antibodies: CD3, rabbit polyclonal (Agilent Technology, Carpinteria, CA, USA), CD204, monoclonal mouse clone SRA-E5 (Cosmo Bio USA, Carlsbad, CA, USA), and FOXP3, rat monoclonal, clone FJK 16s (Waltham, MA, USA). The slides were then incubated for 25 min with the corresponding polymer detection reagent (Biocare Medical). DAB was added to show the sites of antigen–antibody interaction. The slides were then counterstained with hematoxylin, cleared, and cover slipped.

Tumor H&E samples were retrospectively reviewed and graded by a board-certified pathologist. Immunohistochemistry slides were scored on a 1–3 grade based on the percentage of positively stained cells for CD3, CD204, and FOXP3. Grading was determined by calculating the average number of positive cells observed across five distinct fields of 50× magnification. Grade 1 was assigned for an average of 1–5 positive cells, Grade 2 for an average of 5–10 positive cells, and Grade 3 for an average of more than 10 positive cells.

### 2.3. NanoString Tissue Preparation

Tissue tumor sections were deparaffinized in xylene three times for five minutes each, followed by rehydration through sequential immersion in 100% ethanol (twice for two minutes each), 95% ethanol (two minutes), and 70% ethanol (two minutes). The sections were then placed in distilled water until further processing. For lysis, 10 to 50 µL of PKD buffer (Qiagen Inc., Gaithersburg, MD, USA) was applied directly onto the slide. The tissue was scraped off and transferred to a 1.5 mL Eppendorf tube, where Proteinase K (Roche Molecular Systems Inc., Branchburg, NJ, USA) was added at ≤10% of the final volume. The RNA lysate was incubated at 55 °C for 15 min, followed by 80 °C for another 15 min. Quantification was performed with a Qubit Fluorometer (Thermo Fisher Scientific, Waltham, MA, USA), and the RNA lysate was stored at −80 °C until gene expression profiling was conducted using the NanoString nCounter system (NanoString Technologies, Seattle, WA, USA).

### 2.4. Data Analysis

Data analysis was performed using quantile normalization. In this process, the relative ranks of genes (across all genes on the NanoString code set) within each sample were replaced by values having the same relative rank from the pooled distribution (from all samples and genes in the data set). Normalization was executed using nSolver software (version 4.0) (NanoString Technologies). All quantile normalized data subsequently underwent a log10 transformation. Individual genes were compared using an analysis of variance (ANOVA), and genes with a *p* value < 0.05 were included for subsequent unsupervised hierarchical cluster analysis. The individual reduced gene lists for each region were then analyzed in Ingenuity Pathway Analysis (IPA) (Qiagen)(Hilden, Germany) using CORE analysis to identify any significant relationships or associations with known functions or canonical pathways. These predefined networks within the IPA (grouping genes by function, pathway, disease association, etc.) are manually curated from a consortium of published articles and public data. Although this analysis was not used to demonstrate associations between specific genes and STS subtype, it was used to identify potential genes of interest.

Data sets of gene expression obtained from the canine soft tissue sarcomas were compared with a human data set for soft tissue sarcomas [28]. In the human cohort, the most common sarcoma subtype was liposarcoma (33%), followed by pleomorphic undifferentiated sarcoma (25%), leiomyosarcoma (23%), and synovial sarcoma (19%) [28]. A comparative gene data set was created with genes expressed in both human and canine tumor samples. These gene sets covered diverse biological pathways involved in inflammation which have been found to be dysregulated in various cancers. The gene expression normalization was performed using the geNorm algorithm that selected the best housekeeping genes. To visualize the results, unsupervised clustering was used to generate heatmaps based on the QC-passed, normalized data counts of individual genes.

## 3. Results

### 3.1. Patient Demographic

**Figure 1:**
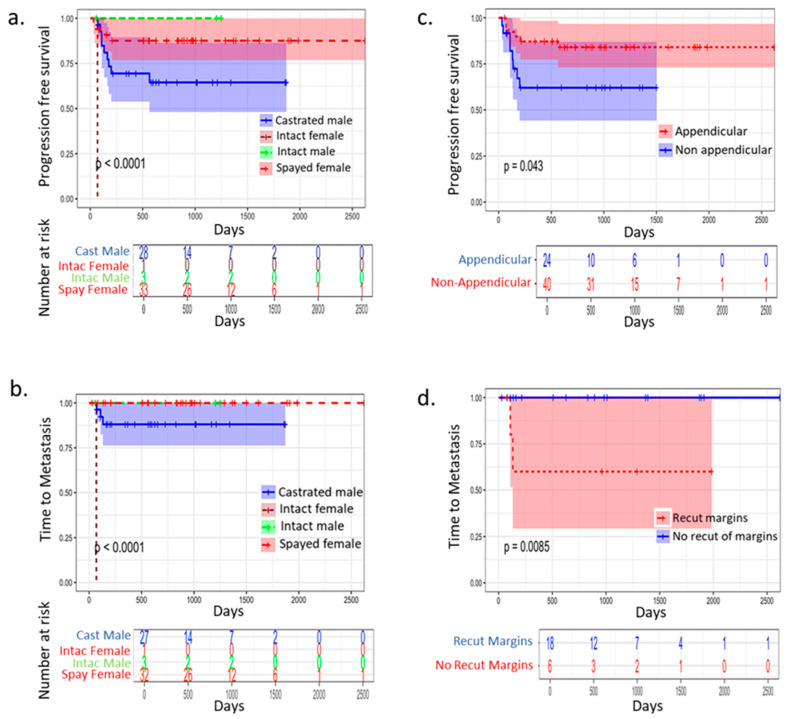
(**a**). A Kaplan–Meier curve illustrating significant differences in progression-free survival among STS dogs based on their spaying and neutering status. Castrated males experienced a significantly shorter time to progression in comparison to spayed females and intact males. (**b**). This curve demonstrates shorter time to metastasis for castrated males when compared to spayed females and intact males. (**c**). Progression-free survival and (**d**). time to metastasis were significantly shorter in dogs undergoing a second procedure for recutting surgical margins regardless of disease grade.

A total of 105 dogs with STS were identified, and of those 75 dogs were included in the analysis. Reasons for exclusion were missing medical records or absence of histopathological diagnosis. Thirty-four breeds were represented with Labrador retriever being the most common, followed by mixed breed dog (Table 1). There were 32 castrated males, 39 spayed females, 3 intact males, and 1 intact female included in the study. Castrated males experienced shorter progression-free survival (Figure 1a) and shorter time to metastasis (Figure 1b) when compared to spayed and intact females. The median age at presentation was 12 years (95% CI: 2–13 years) (Table 1). The tumor location was predominantly appendicular (*n* = 47, 62.66%), and non-appendicular location was diagnosed in 28 dogs (37.44%). There were 29 grade 1 STS (38.66%), 26 grade 2 STS (34.66%), and 20 grade 3 STS (26.66%) (Table 1). Tumor histotype was able to be identified further in 50/75 cases. The most predominant was fibrosarcoma (24%), followed by peripheral nerve sheath tumor (18.66%) (Table 2). Overall, the number of dogs that had metastatic disease at presentation was 6 (8%). Thirteen dogs (17.33%) received pre-operative radiation therapy consisting of five fractions of 5 Gy. Surgical margins following the first surgical resection of the tumors were incomplete in 23 dogs (30.66%).

### 3.2. Outcomes

A total of 14 dogs were treated with adjuvant therapy. Five dogs were treated with radiation following the excision of the primary mass. Adjuvant chemotherapy for soft tissue sarcoma was given to six dogs (doxorubicin, ifosfamide, carboplatin, and intralesional 5-Fluorouracil) (Table 2), and three dogs were treated with chemotherapy for the recurrence of a different cancer (mast cell). Concurrent neoplasia was diagnosed in 24 dogs (32%) (Table 2). In the 16 dogs who had progressive diseases, the median progression-free interval was 120.5 days (95% CI: 31–565 days). Sixteen dogs experienced local recurrence, and six dogs had pulmonary metastatic disease. In patients who were deceased at the time of the study, the median survival time was 299 days (11–1205 days). The median progression-free interval in those without progressive disease at the end of the study was 1048 days (524–2614 days).

Histological grades did not significantly correlate with progression-free survival, recurrence-free survival, or metastasis in this cohort. Spayed females had longer progression-free survival and time to metastasis in comparison to castrated males (Figure 1a,b). Dogs with appendicular location of the primary tumor had a significantly longer recurrence-free survival and progression-free survival than dogs with non-appendicular location (Figure 1c,d). Additionally, patients who required follow-up surgery to resect positive margins due to residual disease experienced a shorter time to metastasis than those who did not undergo additional surgery (Figure 1d).

### 3.3. Immune Microenvironment

**Figure 2:**
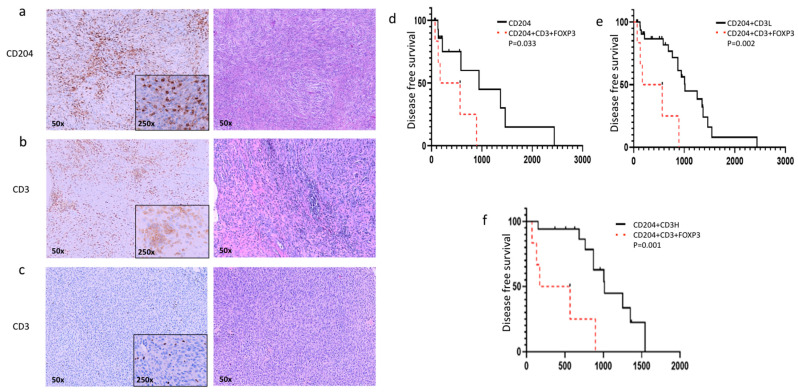
(**a**). Immunohistochemical analysis of STS from three dogs stained with CD204; a common marker for macrophages revealed that most tissue samples in our study exhibited CD204-positive cells with a diffuse distribution throughout the tumor. (**b**). A representative tumor sample showing a high infiltration of CD3 cells, a common marker of T cells, with cytoplasmic and membranous staining of the positive cells (**c**). A Kaplan–Meier curve illustrates a significantly shorter disease-free interval for patients with CD204, CD3, and FOXp3 compared to those with CD204 alone, as well as patients with any CD3^+^ expression (**d**), low CD3 expression (**e**), or high CD3 staining (**f**).

Immunohistochemistry was graded by the average number of CD204 positive cells observed across five distinct fields at 50× magnification (Figure 2a–c). Grade 1 was assigned for an average of 1–5 positively stained cells, Grade 2 for an average of 5–10 positive cells, and Grade 3 for an average of more than 10 positive cells. Immunohistochemistry revealed a high infiltration of immune cells in all the tissues examined regardless of the grade or location of the disease. Excluding four tumors, all samples had infiltration of CD204^+^ cells. Dual positivity for CD204 and CD3 was evident in 26/62 samples. Dogs with FOXP3^+^ cell staining in their tumors had a significantly shorter progression-free interval compared to those without FOXP3 expression (Figure 2d–f).

### 3.4. NanoString Data

**Figure 3:**
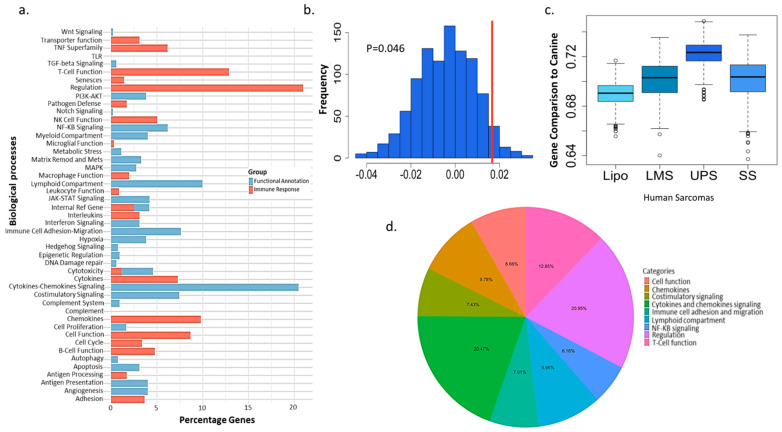
(**a**). Distribution of gene percentages for functional annotation and immune response exhibiting the involvement of key oncogenic pathways such as JAK-STAT, NF-ƘB PI3K. (**b**). Permutation test demonstrating a significant difference between the correlation of human UPS and canine STS in comparison to the human non-UPS tumors and canine STS. (**c**). A comparison between the gene expression of the human and canine gene shows 95% bootstrap closest confidence interval between canine STS and human UPS. Calculation of rank was performed for the common genes between human and canine data and normalized to the log2 NanoString data. (**d**). Proportional distribution of functional gene groups with the most notable one being Regulation (20.95%) and Cytokine and Chemokine Signaling (20.47%).

**Figure 4:**
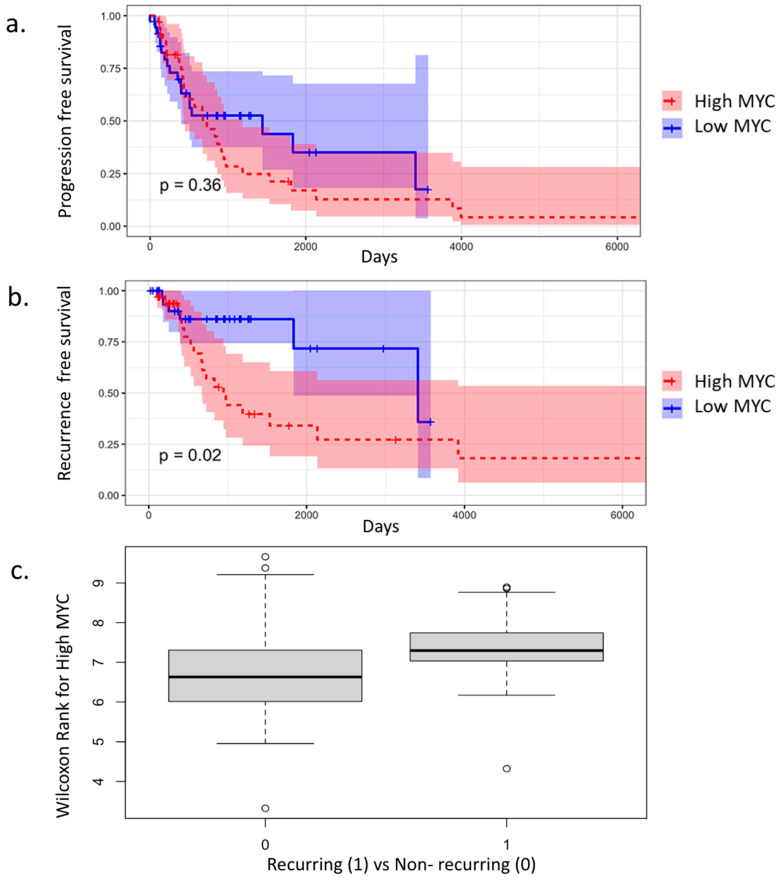
(**a**). Kaplan–Meier curve showing no significant difference in progression-free survival between dogs expressing high or low MYC at the primary tumor. (**b**). This curve shows a significantly (*p* = −0.02) shorter recurrence-free survival for dogs with high expression of MYC in their primary tumor. (**c**). Wilcoxon rank sum test for the high expression of MYC in dogs with recurring disease (1) vs. none (0). The median for the dogs with recurring disease and MYC positivity had a median of 7.3 and was significant.

Analysis of the NanoString data using a permutation test of canine and human STS revealed a statistically significant difference between human liposarcomas and canine soft tissue sarcomas (*p* = 0.008). Canine STS had the most significant correlation to UPS (Figure 3a). A further analysis of UPS and canine STS identified a significant difference in comparison to non-UPS and canine STS (*p* = 0.046) (Figure 3b). The expression of the genes related to the *myc* pathway were significantly expressed in the STS of both species, although the expression of the *myc* gene was not significantly correlated with progression-free survival or time to metastasis in canine (Figure 4a). High levels of *myc* gene expression had a significantly shortened recurrence-free survival regardless of the tumor grade or stage (*p* = 0.02) (Figure 3c and Figure 4b). Wilcoxon rank sum test was performed for the high expression of MYC in dogs with recurring disease (1) vs. none (0). The median for the dogs with recurring disease and MYC positivity had a median of 7.3 and was significant (Figure 4c). Proportional distribution of functional gene groups are shown below, with the most notable one being Regulation (20.95%) and Cytokine and Chemokine Signaling (20.47%) (Figure 3d).

## 4. Discussion

Canine soft tissue sarcomas have an estimated incidence rate of 125 cases per 100,000 dogs annually, compared to 7.1 cases per 100,000 people, allowing a rapid patient recruitment for clinical trials [29,30]. In addition, the biological and clinical similarities between the two species and the reduced regulatory barriers in veterinary research make the canine model ideal for translational studies. Our findings demonstrate the shared factors between the STS of canine and human, and the correlation of these factors with clinical outcome.

In human populations, STS demonstrate higher prevalence in adult males [31]. In this current study, we observed sex-based differences in STS progression. Although the male-to-female ratio in our study was 1:1.5, metastatic disease occurred more frequently in castrated males. Because the overall number of metastatic cases was limited and the cohort included only three intact males and one intact female, a conclusive statement cannot be made and the results should be interpreted with caution. However, the observed trend suggests that sex-linked genetic factors, rather than sex hormones, may influence tumor behavior, given that most dogs in the study were castrated or spayed. Notably, the time to progression was significantly shorter in castrated males compared with both spayed and intact females. This finding should as well be interpreted carefully given the small number of intact animals in the study, but it may offer an additional intriguing indication of a potential role for sex-linked genetic factors in this disease. These outcomes are of particular interest as the routine practice of spay and neuter in veterinary medicine offers a distinctive model for exploring the oncogenic role of hormones and sex-linked genes in STS, which remains challenging in human patients due to ethical and practical limitations.

Tumor location appeared as another parameter that demonstrated a statistically significant association with disease-free interval (DFI) in our study. Specifically, dogs that underwent limb amputation had more favorable recurrence- and progression-free survival times, which is likely due to the complete surgical excision of the primary tumor that often includes histologically clean margins at these locations. In addition, patients who had undergone surgery for recurrent disease showed a higher metastatic rate. This is presumably due to the aggressive biological behavior of high-grade tumors that exhibit a local aggressive behavior with higher metastatic rates, and the potential delay of adjuvant therapy following surgical excision that might contribute to the disease progression. These observations align with findings in human STS, where complete surgical resection is a key prognostic factor for local control and overall survival [5,7].

Histological grade was not a significant prognostic factor in our study. While histological grade is generally considered a major prognostic factor for canine soft tissue sarcomas (STS), its prognostic utility is indeed sometimes limited or inconsistent in certain cases. The grading systems (adapted for dogs) rely on three main criteria: mitotic index, cellular differentiation, and percentage of necrosis [32,33]. Subjectivity in assessing criteria like cellular differentiation and necrosis can lead to disagreement between pathologists, especially for tumors that fall on the border between two grades [34]. The histological grade is a morphology-based assessment, but the true aggressiveness of a tumor is dictated by its underlying molecular and genetic profile. Some tumors might appear as low-grade under the microscope but exhibit aggressive behavior due to genetic mutations or express molecular markers (e.g., high expression of proliferation markers, specific oncogene mutations) that drive early metastasis or resistance to therapy [17]. Conversely, a high-grade tumor might be less aggressive than expected if it lacks certain key molecular drivers. The interaction between the tumor cells and the surrounding non-cancerous cells (e.g., immune cells, fibroblasts) can influence tumor behavior. A low-grade tumor with a pro-tumorigenic TME might behave more aggressively than its grade predicts.

Immunohistochemistry of STS has shown that a diffuse distribution of CD163^+^ tumor-associated macrophages (TAMs) was prominent in all the human sarcomas tested in one study [28]. The expression of CD204, another marker of tumor-associated macrophages (TAMs), has previously been reported in human soft tissue sarcomas (STS) and was detected in all canine tumors examined in the present study. In human STS, co-infiltration of TAMs expressing both CD204 and CD163 at the tumor margins was associated with reduced disease-free survival (DFS), although the overall intratumoral percentage of these cells did not correlate with DFI [28]. While our study did not specifically evaluate CD163^+^ TAMs, the dominant presence of CD204 TAMs suggests a comparable immunosuppressive role within the canine tumor microenvironment, mirroring the immunosuppressive niche described in human. In addition, the expression of FOXp3 in our study (along with CD3 and CD204) was significantly correlated with a shorter DFI in comparison to tumors lacking FOXp3, suggesting a significant role of T-regulatory cells on STS progression.

At the molecular level, comparative gene expression analysis revealed significant dysregulation of several oncogenic pathways in STS tissues of both species. As we demonstrated in this study, one of the most significant findings was the altered expression of the *myc* oncogene. This oncogene plays a central and well-established role in human sarcoma biology, functioning as a master regulator of tumor growth, proliferation, and cellular metabolism. Overexpression of MYC drives a more aggressive tumor phenotype by enhancing cell-cycle progression, promoting genomic instability, and reprogramming metabolic pathways to support rapid tumor expansion. Moreover, MYC dysregulation has been linked to resistance to conventional therapies, including chemotherapy and radiation, ultimately contributing to poorer clinical outcomes for patients [31,35,36,37]. Clinically, aberrant MYC activity is frequently associated with high-grade disease, treatment resistance, and reduced overall survival. Consistent with these observations, in our study the upregulation of MYC in the canine cohort was correlated with a significantly shorter recurrence-free survival (RFS) (Figure 4b), reinforcing the relevance of MYC as a driver of aggressive tumor behavior across species (Figure 4b) [38,39]. The correlation of *myc* and the shorter RSF, independent of disease grade, suggests that this oncoprotein can serve as an independent prognostic biomarker and a potential target for STS patients (Figure 4b).

Other dysregulated pathways shared by both species included PI3K/Akt/mTOR, JAK/STAT, and NF-κB pathways, underscoring the biological similarities of the disease between canine and human (Figure 3a) [40,41]. The role of PI3K/Akt/mTOR pathway in STS has been investigated with early evidence suggesting that its inhibition may yield promising therapeutic outcomes [39,40]. Since Rapamycin, an mTOR inhibitor, has already been evaluated for its safety and pharmacokinetics in canine osteosarcoma, it can be used in comparative STS studies [41]. The molecular targeting of the JAK/STAT pathway was shown to overcome the resistance of STS to Gefitinib, an EGFR inhibitor [29]. EGFR expression has been documented in canine soft tissue sarcomas (STS), and the pharmacokinetics of gefitinib were also previously characterized in canine, supporting the feasibility of a relevant and comparative therapeutic study in this species [40,41].

Undifferentiated pleomorphic sarcoma (UPS) is an aggressive malignancy in humans, characterized by a high rate of metastasis and local recurrence. Interestingly, like soft tissue sarcomas (STS) in canine, UPS commonly arises in the extremities and trunk, with the lungs being the most frequent site of metastasis [42]. A comparison of immune-related gene expression profiles between canine soft tissue sarcomas (STS) and human subtypes revealed the strongest correlation with UPS, followed by synovial sarcoma, leiomyosarcoma, and liposarcoma. These interspecies similarities are further supported by the immune contexture of UPS, which is characterized by increased infiltration of immune cells, including CD3^+^ and FOXP3^+^ T cells, as well as CD204^+^ macrophages mirroring the immune landscape observed in our canine cohort [43,44,45,46,47].

Despite these promising findings, several limitations must be acknowledged. The retrospective nature of this study introduces variability in follow-up durations and uniformity. Additionally, subsets of infiltrating immune cells were not fully characterized, limiting our understanding of their functional roles within the tumor microenvironment. Although there was a suggestive difference in prognosis between castrated males and spayed females, indicating a potentially limited role of hormones, the number of intact canine patients in this study was too small to draw a definite conclusion regarding the impact of hormonal status on prognosis. Finaly, given the limitations of the classification schemes available in canines and the restricted validation of specific IHC stains within this cohort, we were able to classify only 50 of the 75 cases, which narrows the comparative scope of the study.

## 5. Conclusions

In conclusion, this study is a direct comparison of the immune microenvironment in canine and human STS. Our findings showed that the immune landscape of canine STS closely mirrors the disease in humans with high immune cell infiltration, and dysregulation of key oncogenic pathways. We also highlighted the critical immune parameters that influence disease progression and emphasize the potential for targeting immune pathways in both species. These similarities of the immune contexture further validate canine STS as a relevant model for the investigation of novel therapeutic strategies. Future studies should aim to further characterize macrophage phenotypes, expand sample sizes, and explore the therapeutic implications of immune contexture in STS management.

## Figures and Tables

**Table 1:** Patient demographics.

Variable	Number (%)
Total number of patients	75
Median age (years)	12, range (2–13 years)
**Sex**	
Intact male	3 (4)
Intact female	1 (1.33)
Castrated male	32 (42.66)
Spayed female	39 (52)
**Breed**	
Labrador retriever	12 (16)
Mixed breed dog	11 (14.67)
Golden retriever	8 (10.6)
Pitbull terrier	5 (6.66)
Miniature schnauzer	3 (4)
German shepherd	3 (4)
Boxer	3 (4)
Border collie	2 (2.67)
Chihuahua	2 (2.67)
Cocker spaniel	2 (2.67)
Australian shepherd	1 (1.33)
Daschund	1 (1.33)
Rhodesian ridgeback	1 (1.33)
Shiba inu	1 (1.33)
Yorkshire terrier	1 (1.33)
Italian greyhound	1 (1.33)
Great dane	1 (1.33)
Blue tick coonhound	1 (1.33)
Beagle	1 (1.33)
American eskimo	1 (1.33)
Jack Russell terrier	1 (1.33)
Whippet	1 (1.33)
Rottweiler	1 (1.33)
Pembroke Welsh Corgi	1 (1.33)
Black mouthed cur	1 (1.33)
Vizsla	1 (1.33)
Boston terrier	1 (1.33)
English bulldog	1 (1.33)
Dalmatian	1 (1.33)
Cane Corso	1 (1.33)
Husky	1 (1.33)
Beauceron	1 (1.33)
Bernese mountain dog	1 (1.33)
Schnauzer	1 (1.33)

**Table 2:** Tumor descriptions, treatments, and concurrent neoplasia.

Tumor Site	Number (%)
Appendicular	47 (62.67)
Non-appendicular	28 (37.33)
**Tumor grade**	
Grade 1	29(38.6750)
Grade 2	26 (34.67)
Grade 3	20 (26.6789)
**Metastatic disease**	
Nodal Metastases	3 (4)
Lung metastases	5 (6.66)
**Surgical margins following first surgery**	
Incomplete	23 (30.66)
Complete	52 (69.33)
**Adjunctive therapy**	
Pre-operative radiation	13 (17.33)
Post operative radiation therapy	5 (6.66)
Doxorubicin	3 (4)
Doxorubicin and ifosphamide	1 (1.33)
Carboplatin	5 (6.66)
Intralesional 5-FU	1 (1.33)
Chlorambucil	3 (4)
Ifosphamide	1 (1.33)
Palladia	2 (2.66)
**Tumor Histotype**	
Fibrosarcoma	18
Peripheral nerve sheath tumor	14
Perivascular wall tumor	10
Myxosarcoma	1
Liposarcoma	1
Undifferentiated pleomorphic sarcoma	2
Anaplastic Sarcoma	4
**Concurrent Neoplasia**	
Adrenal Tumors	3 (12.5%)
Mast Cell Tumor (Low Grade)	8 (33.33%)
Urothelial Carcinoma	1 (4.16)
Perianal Adenoma	1 (4.16)
Hepatocellular Carcinoma	2 (8.33%)
Splenic Nodules	2 (8.33%)
Cutaneous Hemangiosarcoma	1 (4.16)
Intramuscular Hemangiosarcoma	1 (4.16)
Trichoepithelioma	1 (4.16)
Brain Tumor	2 (8.33%)
Other Sarcoma	2 (8.33%)

## Data Availability

The data sets generated during and/or analyzed during the current study are available from the corresponding author on reasonable request.
